# Effect of Straw Provision in Racks on Tail Lesions, Straw Availability, and Pen Hygiene in Finishing Pigs

**DOI:** 10.3390/ani11020379

**Published:** 2021-02-02

**Authors:** Torun Wallgren, Stefan Gunnarsson

**Affiliations:** 1 Department of Animal Environment and Health, Swedish University of Agricultural Sciences, P.O. Box 70568, 750 07 Uppsala, Sweden; 2 Department of Animal Environment and Health, Swedish University of Agricultural Sciences, P.O. Box 234, 532 23 Skara, Sweden; stefan.gunnarsson@slu.se

**Keywords:** tail biting, fattening pig, swine, welfare, fattener, tail docking

## Abstract

**Simple Summary:**

Pigs spend the majority of their time exploring their surroundings. Unfulfilled exploratory behavior has been linked to tail biting in pigs, leading to decreased welfare and production. Straw provision enables exploratory behavior and reduces tail biting, but large amounts of straw may be difficult to incorporate into current production systems, causing e.g., poor hygiene. This study examined whether provision of straw in racks, rather than on the floor, can enable larger straw rations without compromising hygiene. The study was conducted on a commercial farm with 458 undocked pigs in 42 pens provided with straw in racks or on the floor. Available straw and manual cleaning requirement were assessed daily, and presence of tail lesions was assessed weekly. Compared with pigs in the floor treatment, pigs in the rack treatment had more lesions in the beginning of the production period, but fewer tail lesions at the end. This could be because pigs in the rack treatment initially did not consume straw from the rack, leading to low straw access early in the production period.

**Abstract:**

Unfulfilled exploratory behavior in pigs has been linked to tail biting, which causes reduced performance and welfare. Provision of straw can reduce tail biting, but large straw rations can cause poor hygiene in pens. This study examined whether provision of straw in racks, rather than on the pen floor, can enable larger straw rations without compromising hygiene. The study was conducted on a commercial farm with 458 undocked pigs in 42 pens provided with straw in racks or on the floor. Available straw and manual cleaning requirement were assessed daily, and presence of tail lesions was assessed weekly. Both treatments had a low requirement for manual cleaning (Floor: 1.7%, Rack: 1.8%). Pigs in the rack treatment had a higher incidence of lesions early in the production period, which coincided with these pigs initially not consuming straw from the rack, leading to low straw access. Late in the production period, these pigs had learned how to use the rack and had a lower incidence of lesions than pigs in the floor treatment. Delayed use of the rack may have been linked to undeveloped spatial skills in the pigs, which needs further research.

## 1. Introduction

Pigs are highly active animals, spending the majority of their active time investigating their surroundings in a search for, e.g., feed and sleeping places [1,2,3]. Pigs reared in barren environments, unable to express their natural behavior, are known to redirect their exploratory behavior towards pen fittings and other pigs, which can lead to tail biting [4]. Tail biting, defined as one pig orally manipulating the tail of another pig, is an animal welfare issue for several reasons [5,6]. In the bitten pig, tail biting causes pain and reduced health, growth, and production profits [7,8,9]. For the biting pig, the behavior is a sign of stress and of unfulfilled behavioral needs [10]. Tail docking is a commonly used procedure to minimize the impact of tail biting, but is prohibited by EU legislation (Council Directive 2008/120 EC) and only treats the symptoms, and not the underlying cause [10,11,12,13,14,15]. In order to rear pigs without risking tail biting or having to perform the unethical treatment of tail docking, the behavioral needs of pigs must be fulfilled to the extent that exploratory behavior is not redirected to other pigs, or at least kept to a minimum.

One established means to prevent the development of tail biting is provision of straw, which enables exploratory behavior [8,16,17,18]. The amount of straw needed to fully meet the behavioral needs of the pig is reported to be approximately 500 g per pig and day [19]. However, smaller amounts of straw, from 10 g per pig and day, can also reduce the occurrence of tail biting [16,20,21]. In pigs reared without tail docking, low prevalence of tail lesions at the abattoir is currently achieved partly by the use of straw, commonly provided on the floor [20,21]. A potential drawback with the use of straw is poor compatibility with current production systems, such as difficulties in providing straw on fully slatted floors and the negative impact of straw on pen hygiene [4,13,22,23]. In previous studies, we demonstrated that straw provision does not lead to poor pen hygiene [20,21,24]. However, the large amounts of straw suggested to be necessary in order to fulfill the behavioral needs of the pigs [19] can be difficult to supply in current pig barns, where manure handling systems are designed for low throughput of straw [20].

The overall aim of this study was to improve animal welfare by identifying different ways of providing pigs with straw in order to reduce tail biting. The specific objective was to investigate whether provision of straw in racks could be a way of providing larger amounts of straw, and thereby enabling more explorative behavior and minimizing tail biting in finishing pigs. The effect of straw provision on pen hygiene was also investigated, in order to assess functionality in production systems with partly slatted floors. Additionally, straw availability (permanent access to straw) in the treatments with straw supplied in racks or on the floor was investigated. The effects of the treatments on tail damage and tail length were also investigated. The hypothesis was that an increased straw ration provided through straw racks increases straw availability to the pigs over time compared with provision of straw on the floor.

## 2. Materials and Methods

The general aim was to improve animal welfare by investigating different ways of providing pigs with straw in order to reduce tail biting. The study comprised behavioral observations and clinical scoring of pigs on a commercial pig farm, along with provision of extra enrichment (straw) without any invasive treatment. Thus, ethical approval by an ethics committee for animal experiments was not required according to Swedish Legislation (SJVFS 2019:9 Case no L 150. The Swedish Board of Agriculture’s Regulations on Research Animals; The Swedish Board of Agriculture: Jönköping, Sweden, 2019). Sweden is part of the European Union. All pigs were managed and treated according to normal management routines by staff at the commercial farm where the intervention study was performed.

### 2.1. Animals and Housing

The study was conducted on a commercial farrow-to-finish pig farm in southwest Sweden. All pigs were progeny of crossbred sows (Landrace × Yorkshire (TN70) and Hampshire boars). No pigs were tail docked, as the practice is banned under Swedish national legislation (§2 4 Chap (2018:66)). Males were surgically castrated during the first week of life, after analgesic treatment (0.3–0.5 mL Lidokel-Adrenalin vet^®^, KELA N.V, Hoogstraten, Belgium). The piglets were weaned at approximately five weeks of age and transferred to a weaner unit, where they were housed in similar groups as in the finishing pig unit meaning that the pigs were commonly kept in the same groups but occasionally regrouped if necessary, i.e., to have the same number of pigs in each group or removal of sick pigs. Straw was provided to the pigs at all stages of production.

**Figure 1:**
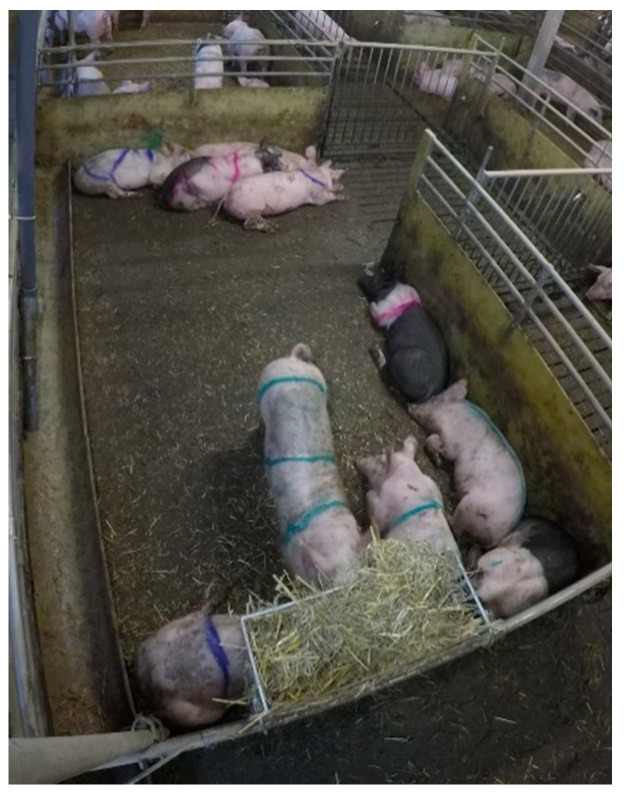
Pen design, including location of the straw rack.

The experiment was conducted with one batch (*n* = 459) of finishing pigs from approximately 30 kg live weight (LW) until slaughter at around 120 kg LW. The experiment started as the batch of pigs entered the finishing pig compartment and ended as the majority (>70%) of the pigs were sent to slaughter after 14 weeks (102 days). As the pigs were moved into the barn, they were partly sorted by size (keeping the largest pigs together and the smallest pigs together, grouped to minimize the risk that pigs of a certain size were over represented in a specific treatment group), but not by sex, into 42 pens. Each pen housed 10 (*n* = 4, 1.05 m^2^/pig), 11 (*n* = 37, 0.95 m^2^/pig), or 12 (*n* = 1, 0.87 m^2^/pig) pigs and had a partly slatted floor (total area 10.49 m^2^, slatted area 2.68 m^2^) (Figure 1). Each pen contained a 3.4 m long feeding trough at the long side of the pen, and one nipple drinker in the slatted area. The pigs were fed a cereal-based liquid feed, through an automatic feeding system (4 times/day until week 12, 3 times/day week 13–14). The pigs were inspected daily and the solid section of pen floor was cleaned manually.

As the pigs were not individually ear-tagged, manual marking with spray paint (PORCIMARK^®^ marking spray, Kruuse, Langeskov, Denmark) was carried out twice a week in order to keep track of individuals. The pigs were marked with 1–3 lines on their back, in green, blue, or red (Figure 1). One pig per pen was left unmarked.

### 2.2. Experimental Set-Up

**Figure 2:**
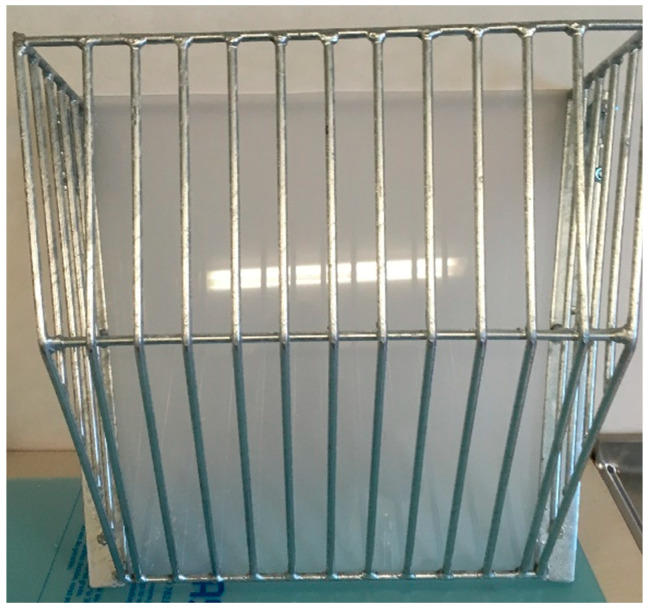
Modified straw rack from JYDEN (3620-2012). The modification consisted of a plastic back-plate. The rack was fitted on the pen wall with pipe clamps.

All pens were treated the same apart from the fact that straw was provided either on the floor (Floor treatment) or in a straw rack (Rack treatment). During daily management, the rack in pens with a straw rack was refilled daily, with up to ~44 L of straw. Pens without a straw rack were provided with around 25 L straw on the solid floor of the pen (approximately 1.8 kg) daily. In order to minimize the effect of pen location in the house (due to microclimate etc.), alternate pens were equipped with a straw rack or provided with straw on the floor. The straw rack used was from JYDEN (JYDEN, Saeby, Denmark, art. No. 3620-2012) and was modified with a Plexiglas back-plate in order to fit the pen (L 50 cm, H 50 cm, W 21 cm; 30 mm between bars) (Figure 2).

Leftover straw. Straw was provided daily. Before provision of new straw, the amount of leftover straw (i.e., clean straw on the floor or in the rack) was assessed by the caretaker (Table 1). If the straw rack was full, no new straw was provided.

Pen hygiene. Assessment of pen hygiene was done daily, by recording of pen cleaning events. Before provision of straw, the caretaker cleaned the pen manually if needed, i.e., removed soiled straw or manure from the solid floor.

Tail lesion scoring. The tails of the pigs were scored once per week (Time, 14 occasions) through palpation and assessing tail length and tail damage on individual level (Table 2). Pigs that were observed to be severely limping, unwilling to stand, or unwilling to put weight on at least on limb were scored lame. All scoring was carried out by the same operator.

### 2.3. Statistical Analysis

Results were recorded on paper and later transferred into Microsoft Excel. Data were statistically analyzed using SAS 9.4 (SAS Inst. Inc., Cary, NC, USA). Descriptive analyses of tail lesions, development of tail damage, and leftover straw were performed using PROC MEANS and PROC FREQ.

Tail lesions. As the lesion scoring was ordinal and not normally distributed tail lesions were converted into binomial traits in the analysis. This meant that the either the pig was scored with a lesion of a specific grade or not. Lesions were recorded with regard to tail length (L1–2, compared to L0; L2 Compared to L0–1) and tail damage (D1–4, compared to D0; D2–3, compared to D0–1; D3–4 compared to D0–2; D4 compared to D0–3). In order to analyze the effect of treatment (Floor, Rack) on tail lesion scoring, analysis of variance (PROC GLIMMIX) was used to construct a statistical model for each trait described (tail length, tail damage). All statistical analyses of lesion scores were performed with pig as the experimental unit. The statistical model included the effects of treatment (Floor, Rack), time (1–14), sex (Gilt, Castrate, Boar), and the interaction between treatment and time. Nonsignificant effects were removed from the model. Pig (within pen) was considered a random effect, and consideration was taken of repeated observations within pig. Least square means were used to compare treatment effect at the different time points.

Development of tail damage. Data was checked for normality, using proc univariate. In order to analyze the development of tail lesions, the difference in lesion scores between two adjacent score measurements was calculated (Difference_damage_ = Damage_TimeX_ − Damage_TimeX+1_). Difference_damage_ values were then analyzed in order to investigate the impact of treatment on lesion development. The data was sufficiently normally distributed in order to fit a robust model. To analyze treatment effects (Floor, Rack) on Difference_damage_, analysis of variance (PROC MIXED) was used to construct a statistical model for each trait analyzed (damage change). All statistical analyses of damage development were performed with pig as the experimental unit. The statistical model included the effects of treatment (Floor, Rack), time, sex, and the interaction between treatment and time. Nonsignificant effects were removed from the model. Pig (within pen) was considered a random effect and consideration was taken of repeated observations within pig.

Leftover straw. In order to investigate the effect of treatment (Floor, Rack) on leftover straw, the mean amount of left over straw per treatment group were plotted over time (days in production).

## 3. Results

Five pigs were removed from the study for other reasons than slaughter. One was found dead without previous signs of sickness, two pigs were euthanized due to sickness (unspecified), and two pigs were removed due to severe lameness. All removed pigs were from the straw rack treatment.

### 3.1. Tail Lesions

**Figure 3:**
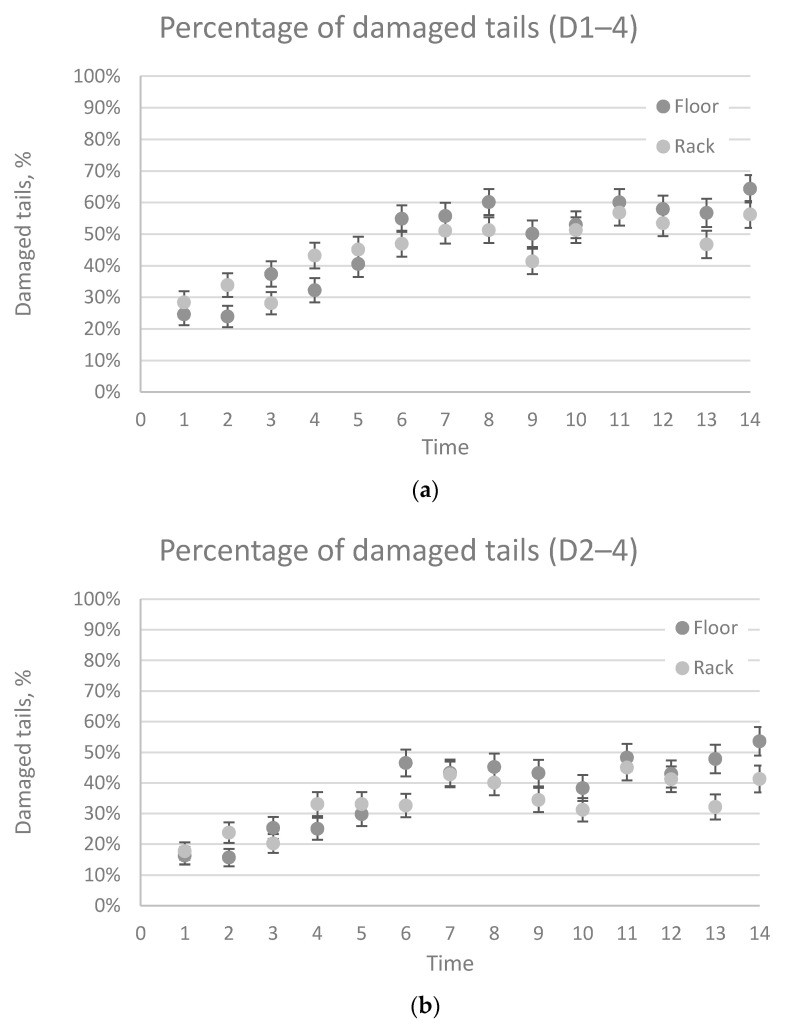

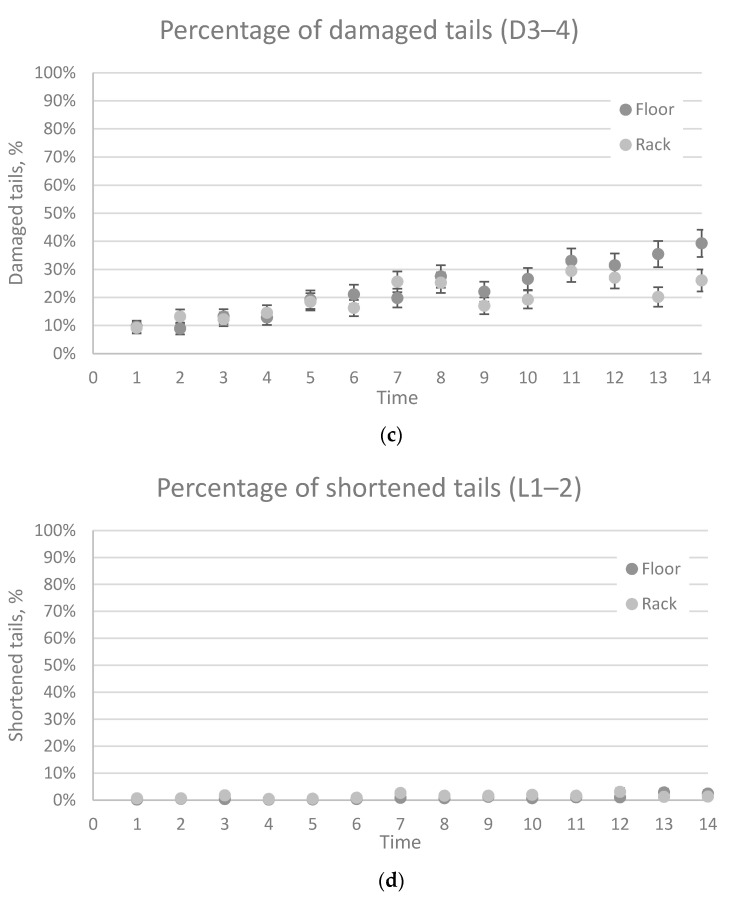
Interaction between percentages of pigs with damaged tails and time (**a**–**c**), percentage of shortened tails and time (**d**). Time refers to the time of data collection, which was done once per week during the production period (1–14). The error bars are standard error means and visualizes where there is a difference between the two treatments. (**a**) Percentage of damaged tails with damage score D1–D4, (**b**) percentage of damaged tails D2–4, (**c**) percentage of damaged tails D3–4, and (**d**) percentage of shortened tails (L1–L2). For explanation of damage and length scores, see Table 2. N_tot_ = 6139, N_Time1_ = 459, N_Time2_ = 458, N_Time3_ = 455, N_Time4_ = 451, N_Time5_ = 541, N_Time6_ = 452, N_Time7_ = 450, N_Time8_ = 448, N_Time9_ = 448, N_Time10_ = 447, N_Time11_ = 433, N_Time12_ = 433, N_Time13_ = 377, N_Time14_ = 376.

On average, 3.7% (1.4% at first observation) of pigs in the Rack treatment and 5.9% (5% at first observation) of pigs in the Floor treatment had tail length score 1. A maximum of one pig per treatment had a score of 2 for tail length at any time point. The amount of damaged tails increased over time, and was approximately 25–30% at the first scoring and 55–65% at the last scoring (Figure 3a). Treatment or sex had no effect on tail damage or tail length, while time had a significant effect on all tail damage and tail length (damage 1–4, *p* < 0.0001, damage 2–4 *p* < 0.0001, damage 3–4 *p* < 0.0001, tail shortening *p* < 0.0001, Figure 3). The interaction between treatment and time was significant for damage traits 2–4 (*p* = 0.0022). The interaction between treatment and time was also significant for damage 1–4 (*p* = 0.0054) and close to significant for damage 3–4 (*p* = 0.0581), Figure 3). The effect of treatment on tail damage 4 or tail length 2 could not be investigated further, due to the low amount of pigs scored with tail length 2 (0.37%) or damage 4 (1.4%). The farm personnel reported tail biting in 0.19% of the Rack pens and 0.14% of the Straw pens.

### 3.2. Change in Tail Damage

**Figure 4:**
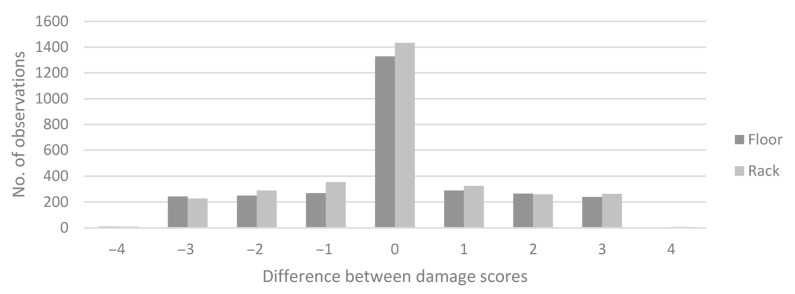
Difference in tail damage between two adjacent weekly scorings of tail damage on the same pig in the Floor and Rack treatments. *n* = 6053.

**Figure 5:**
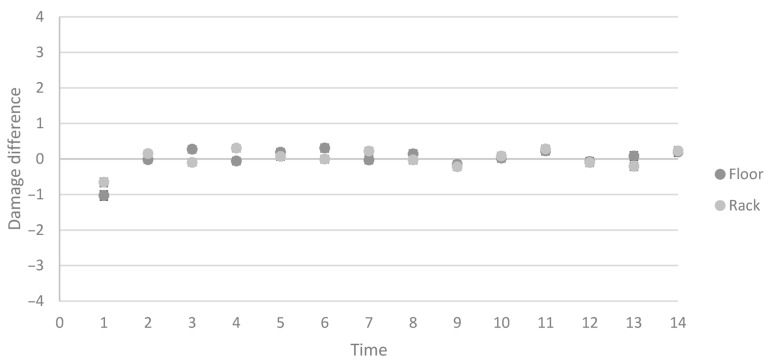
Estimated mean tail damage difference over time (once per week during the production period) in the Floor and Rack treatments. The error bars are standard error, visualizing when there is a treatment effect. *n* = 6053.

The change in tail damage ranged from −3 to +3 between scorings, but most commonly the tail damage score did not change between two adjacent scorings (Figure 4). Treatment or sex had no effect on the change in tail damage, while time (*p* < 0.0001) and the interaction between treatment and time (*p* = 0.0144) had significant effects (Figure 5).

### 3.3. Leftover Straw and Pen Hygiene

**Figure 6:**
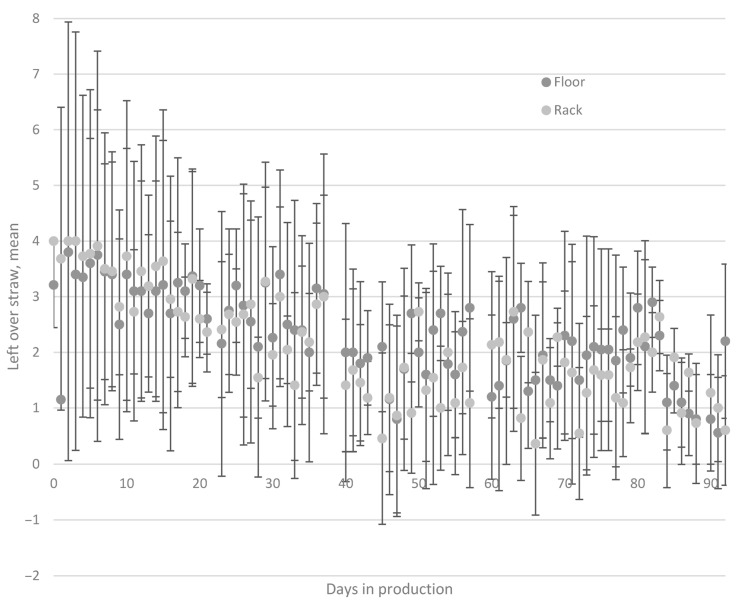
Mean amount of leftover straw and mean standard deviation, in the pen on the daily inspection during the production period. Leftover straw was measured on the floor in the Floor treatment and in the rack in the Rack treatment. *n* = 2978.

Mean amount of leftover straw varied over time and was higher in the beginning of the study period (Figure 6). Manual cleaning was performed on 1.8% of daily inspection occasions in Rack and 1.7% of occasions in Floor. The farm personnel suspended straw provision on 3.3% of daily inspection occasions in Floor and 12.3% in Rack. The main reason for suspending straw provision in Rack was because the rack was still full (i.e., the pigs had not emptied the rack). When removing the observations where new straw was not provided due to the straw rack already being full, the percentage of occasions on which new straw was not provided in Rack (e.g., due to poor pen hygiene) was 0.64%. In Floor, 4.3% of the observations were scored as 0, 14.3% as 1, 34.7% as 2, 31.8% as 3, and 14.9% as 4. In Rack, 24.7% of the observations were scored as 0, 10.2% as 1, 11.2% as 2, 22.0% as 3, and 31.8% as 4.

## 4. Discussion

Tail biting and the resulting tail lesions have been linked to unfulfilled exploratory behavior in pigs [3]. It is known that provision of straw enables exploratory behavior, which can reduce tail lesions [11,25]. This study investigated whether straw racks could enable larger straw rations, and, hence, reduce tail lesions compared with straw provision on the floor. There was no “pure control”, i.e., one treatment entirely without straw provision, in the study, because rearing pigs without provision of material enabling proper investigation and manipulation is forbidden under the EU Pig Directive (Council Directive 2008/120/EC, 1 Chap §4) and Swedish national legislation (SJVFS 2019:20 4 Chap §4). Further, rearing pigs in a barren environment is related to the development of tail biting, which would have posed a risk of unnecessary suffering for the pigs in this study. The results showed that provision of straw in racks had no significant effect on tail lesions or tail damage development compared with straw provision on the floor. However, number of tail lesions observed increased with increasing pig age, which is in line with previous studies [5,21].

There was an interaction between treatment and time, revealing that type of straw provision had an effect on tail lesions on several occasions. Damaged tails were more common in pigs receiving straw in racks in the beginning of the production period (Damage 1–4, Time 2, 4; Damage 2–4, Time 2, 4), while damaged tails were more common in pigs that received straw on the floor in later stages of the production period (Time >6).

Tail status commonly remained the same between two adjacent soring sessions (one week apart), but sometimes differed by up to four steps (either from most severe score to undamaged or the other way round) although it was less common. This implies that tail biting damage can escalate quite quickly (−4), but also that tail lesions can heal within a week (+4) if conditions are right. However, tail lesions commonly remained the same over time, indicating that tail biting behavior was consistent over the period (i.e., the damage outcomes were constant). It should, however, be noted that in the present study, there were very few observations of severe tail damage (score 4) and that most damages did not include skin puncture (score 1–2). The healing process for more severe tail damage is longer. In this study, there were also very few observations of tail shortening, especially severe shortening of the tail. Shortening of the tail always include heavy damage and tissue loss, which should increase the healing process further. The current results should, therefore, be considered a reflection of the amount of tail biting and type of tail damages represented in this specific data set. In future research, it would be interesting to investigate whether tail biting interventions (such as tar on tail, extra straw, NSAID, etc. [20]) have a positive effect in reducing tail lesions.

It was hypothesized that straw racks could enable larger straw rations and increase straw availability. The results revealed a significant treatment x time effect, with pigs in the Rack treatment having more tail damage in the beginning of the production period (Time 2–4) and pigs from the Floor treatment displaying more tail damage later in the production period (Time 6–14). A possible reason for this could be that pigs in the Rack treatment did not empty the rack in the beginning of the production period (based on the leftover straw scores), so a limited amount of straw was available for the pigs in that treatment. Therefore, the treatment effect in the beginning of the production period may reflect the fact that straw was unavailable for the Rack pigs, and, hence, exploratory behavior was not fulfilled, leading to more redirected tail biting behavior. Later on in the production period, as the pigs started to consume straw from the straw racks, a treatment x time effect indicated less tail damage in the Rack treatment. In the last two weeks of the study, pigs in the Rack treatment showed fewer bite marks and lesions on the tails. This probably reflects the fact that straw was more available in the Rack pens by that stage, hence, enabling more exploratory behavior. As pigs started to empty the straw racks, those in the Rack group were provided with more straw compared to the Floor group. This means that the pigs in Rack also had a greater access to straw compared to Floor (25 L vs. 44 L). In Rack, there was a higher proportion of “extreme” scorings (4 and 0) compared to Floor. This also indicates that the pigs in Rack did either not interact with the straw rack at all (score 0) or emptied the rack (score 4) in approximately 50% of the occasions. Since the left over straw on the floor was not measured in the rack treatment, the effect of straw accessibility when the rack was emptied could not be investigated.

Straw provision, and especially in larger amounts, has previously been pointed out as a potential risk factor for poor pen hygiene [4,22]. Soiled straw in the solid area of the pen may lead to poor pen hygiene and subsequently poor pig hygiene. In this study, the pen hygiene was investigated through amount of manual cleaning as a sign of poor hygiene in the pens, as soiled straw needs to be manually removed from the pen. The caretakers made daily notations regarding the need for manual cleaning of the pen. The amount of manual cleaning were similar between the two groups 1.7% in Floor and 1.8 in Rack), which indicates that the Treatment did not have an effect on the hygiene of the pens. Further, the infrequent amount of manual cleaning indicates that poor hygiene is not a large problem within these management methods. The caretaker could also make interruptions in the straw provisions, which could be due to poor pen hygiene. Interruption of straw provision was done 0.64% of the times in Rack (when removing observations of straw not being provided due to the fact that the straw rack was already full) and 3.3% in Floor. This further implies that hygiene is not a large issue within any of the treatments. Numerically, it further indicates that the Rack treatment were less subjected to poor hygiene, although we know that the pigs in the Rack treatment did not access the straw during parts of the production period, which may have reduced the impact of straw on pen hygiene. The findings that straw, in either treatment, did not have a negative effect on hygiene are in line with previous studies under similar rearing conditions [20,21,24].

The Rack pigs did not start to interact with the straw rack and use the straw immediately, as seen in the amount of leftover straw, which was an unexpected finding. All pigs in the study had access to straw from birth and throughout the nursery and grower period, and were, thus, very accustomed to straw, so we presumed that they would access the straw rack immediately, reflecting spatial thinking. Development of spatial thinking has been investigated in other species (e.g., rats, laying hens). In rats, it has been shown that environmental enrichment, such as environmental complexity, enhances performance of spatial tasks, and that rats raised under conditions with more environmental complexity are more adaptable to changes in context [26]. In laying hens, it has been shown that chicks not provided with a perch at a young age have a higher prevalence of floor eggs and cloacal cannibalism, possibly due to undeveloped spatial skills and, hence, reduced capability to exploit three-dimensional spaces [27]. Early access to perches has been shown to reduce the prevalence of floor eggs and cloacal cannibalism [28]. For the pigs in the present study, prior to the experiment all straw was provided on the floor. Apart from the nipple drinker, the pigs had previously never been able to interact with anything other than on floor level, so their spatial development may have been restricted. The current Swedish legislation requires, e.g., laying hens to be reared in the same type of environment as they will encounter during the productive state (SJVFS 2019:23 2 Chap, §2), e.g., layers in furnished cages should be reared in cages, in order to allow the birds to thrive in their production environment. The fact that the pigs manage to learn to use the nipple drinker, proving that they have spatial ability enough to use a device above floor level, while not immediately learning to empty the straw rack could possibly be affected by two things; instant reward and motivation. Spatial learning is commonly tested through tasks or mazes where animals search for rewards. The most efficient strategy is then to only visit locations that contain rewards [29]. In spatial memory tasks, animals must, therefore, remember unrewarded visits [29]. The initial visits to the straw rack may very well have been unrewarding, if the pigs were unable to retract straw from the rack and did, hence, not motivate the pigs to try again. The height of the straw rack in relation to the pigs’ height could also have had an impact on the pigs’ utilization of the straw racks. In the beginning of the experiment, the pigs weighed around 30 kg. At 30 kg live weight, a pig is around 44–47 cm in withers height. The straw rack was mounted around 50 cm above the ground, which means that in the beginning of the production period the pigs were only able to interact with the lower part of the rack, which might have reduced their possibility to interact with and empty the straw rack. The pigs were, however, able to reach the rack throughout the experiment. Compared to the nipple drinker, which is designed to easily provide an instant reward (water), the straw rack might provide with a higher level of difficulty in the beginning. Further, it could be assumed that the motivation to consume water is higher compared to the motivation to access straw, especially as the pigs do not need to consume the straw for nutritional value. The spatial development of pigs and the impact of experiences from previous environment need more research, in order to establish whether straw racks should be provided during rearing, or in a different manner in order to be fully utilized by pigs.

Potential fear of a novel device of the rack was not considered. However, the rack was installed before the pigs entered the stable and the entire stable was novel to the pig, and we did not detect any specific fear against the straw rack. No test to test the interaction or novelty of the rack was, however, conducted, while habituation to the rack can be assumed to have occurred after some time. The fact that the rack was filled with straw, of which the pigs were accustomed to since birth, should have had a positive impact on the habituation of the straw racks.

We found no effect of treatment on tail length, i.e., tail shortening was equally frequent in both treatment groups. This is probably because the total number of pigs displaying tail shortening was very low, and, hence, there was too little variation within the data to reveal any significant differences. In previous studies applying similar conditions, tail shortening in pigs was also rare [21]. Tail biting is a multifactorial issue, and the fact that the amount of severe tail damage or tail shortening was relatively low regardless of treatment was probably related to the overall housing and management of the pigs apart from the addition of straw. On a positive note, the pigs had a generally high weaning age, around five weeks compared to three weeks, which is common within the EU [30]. Further the stocking density and group size was relatively low, compared to EU [15,30]. The stable groups are also considered positive for tail biting [31]. The partly slatted flooring, in contrast to fully slatted floors have been considered positive for tail biting, enabling manipulation of straw on the floor and a more comfortable lying area [30]. Combined, these factors have been described important for the successful rearing of pigs with intact tails in Sweden [30]. The management and housing was also associated with some risk factors for tail biting, such as liquid feeding and automatic feeding [15].

Overall, with the thorough scoring scheme applied, many pigs (increasing from around 20 in the beginning of the production period to around 65% in the end of the production period) had some form of tail damage. However, it should be kept in mind that most of the tail damage recorded would likely not have been considered serious enough to be scored as tail damage at, e.g., carcass inspection at the abattoir. During scoring, each tail was manually palpated and visually inspected for any signs of tail damage, including damage not puncturing the skin (damage scores 1 and 2), and no distinction was made regarding the size of lesions that punctured the skin (damage scores 3 and 4). The majority of the tail damage described as skin lesions (damage scores 3 and 4) comprised lesions less than 5 mm long, which would not have been recorded as tail damage by the farmer or the abattoir. This is confirmed by the low percentage of times the farm personnel reported tail damage, as found in previous research [32,33]. The mean incidence of tail damage registered at Swedish abattoirs is around 2% [34]. A pig is registered as tail-bitten or having tail damage at the abattoir when at least half of the tail is missing or when signs of tail damage are evident in the official control [35]. However, the percentage of pigs with shortened tails corresponds better to the prevalence of pigs scored as tail-bitten at the abattoir. As the damage-scoring scheme in this study was more detailed, especially regarding milder lesions, it provides a more comprehensive description of pig tail damage. The majority of the tail damage observed in this study might not impair health status or growth rate of the pig directly, but is a sign of unwanted behavior and should be acted upon.

## 5. Conclusions

Provision of straw in racks, compared with on the pen floor, decreased tail lesions in finishing pigs during the latter part of the production period. However, pigs provided with straw on the floor had fewer tail lesions early in the production period. Compared to the end of the production period, the pigs did not empty the straw racks in the beginning of the production period. Hence, access to straw coincided with a lower incidence of tail lesions. The use of straw racks may be a way of providing pigs with more straw under current production systems, without jeopardizing the throughput capacity of the manure system. However, more knowledge is needed regarding use of straw racks by pigs and the impact of early experiences and spatial development.

## Figures and Tables

**Table 1:** Scoring system used to assess leftover straw on the floor and in straw racks (modified from Pedersen et al., 2014 [19]).

Score	Description	Rack Equivalent
0	<0.1 L straw	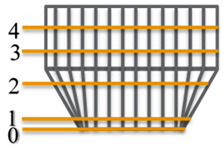
1	0.1–1 L straw	
2	1–10 L straw	
3	10–20 L straw	
4	>20 L straw	

**Table 2:** Scoring system used to assess tail length (L0–L2) and tail damage (D0–D4) [21]

	Score 0	Score 1	Score 2	Score 3	Score 4
Length	L0	L1	L2		
	No shortening.	Part of the tail tissue bitten off, tail shortened to length >2 cm.	Part of the tail tissue bitten off, tail shortened to length <2 cm.		
Damage	D0	D1	D2	D3	D4
	No visible damage.	Tail red and/or swollen. Tail has no bite marks, skin on the tail is not broken.	Tail has bite marks, seen as small red/black dots on the tail, from bruising without broken skin or small holes in the skin, but no missing tissue.	Tail has one or more open wounds, from scratches (without blood) to shortened tail with deep wound (with blood). Wounds can have an intact or partly detached crust.	Tail is swollen and has one or more open wounds, from scratches (without blood) to shortened tail with deep wound (with blood). Wound can have an intact or partly detached crust.

## Data Availability

The data presented in this study are available on request from the corresponding author. The data are not publicly available due to privacy reasons.
